# Clearance of Pneumococcal Colonization in Infants Is Delayed through Altered Macrophage Trafficking

**DOI:** 10.1371/journal.ppat.1005004

**Published:** 2015-06-24

**Authors:** Steven J. Siegel, Edwin Tamashiro, Jeffrey N. Weiser

**Affiliations:** 1 Department of Microbiology, University of Pennsylvania, Philadelphia, Pennsylvania, United States of America; 2 Department of Ophthalmology, Otorhinolaryngology, and Head and Neck Surgery, Ribeirao Preto School of Medicine, University of Sao Paulo, Sao Paulo, Brazil; 3 Department of Pediatrics, University of Pennsylvania, Philadelphia, Pennsylvania, United States of America; The University of Alabama at Birmingham, UNITED STATES

## Abstract

Infections are a common cause of infant mortality worldwide, especially due to *Streptococcus pneumoniae*. Colonization is the prerequisite to invasive pneumococcal disease, and is particularly frequent and prolonged in children, though the mechanisms underlying this susceptibility are unknown. We find that infant mice exhibit prolonged pneumococcal carriage, and are delayed in recruiting macrophages, the effector cells of clearance, into the nasopharyngeal lumen. This lack of macrophage recruitment is paralleled by a failure to upregulate chemokine (C-C) motif ligand 2 (*Ccl2* or *Mcp-1*), a macrophage chemoattractant that is required in adult mice to promote clearance. Baseline expression of *Ccl2* and the related chemokine *Ccl7* is higher in the infant compared to the adult upper respiratory tract, and this effect requires the infant microbiota. These results demonstrate that signals governing macrophage recruitment are altered at baseline in infant mice, which prevents the development of appropriate innate cell infiltration in response to pneumococcal colonization, delaying clearance of pneumococcal carriage.

## Author Summary

Infants are particularly susceptible to infections, though why is not well understood. One important cause of infant mortality worldwide is infection with *Streptococcus pneumonia*e, the pneumococcus. All pneumococcal disease begins with asymptomatic colonization of the upper respiratory tract. Infants are also more likely to carry pneumococci, and on average each carriage event has a longer duration. Here, we used an infant mouse model of pneumococcal colonization to study the mechanisms underlying delayed clearance of carriage. We found that infant mice were unable to recruit the effector cells of clearance, macrophages, into the lumen of the upper airway, and that this delay was accompanied by an inability to produce a macrophage chemoattractant in the nasopharynx. We attribute this defect to a dysregulation in the expression of these chemokines and show this effect results from the commensal bacterial flora of infants. Our findings provide an explanation for why infants are more susceptible to being colonized with and infected by pneumococci.

## Introduction

Many infectious diseases target infants, although our understanding of the host factors that contribute to the increased susceptibility of early childhood remains incomplete [1]. Prominent among the causes of infection of the infant period is *Streptococcus pneumoniae*, the pneumococcus. Despite effective antibiotics and vaccines, pneumococci are responsible for more than 1 million deaths annually, predominantly in the developing world [2]. Worldwide, this gram-positive bacterium is a common cause of pneumonia at all ages. The spectrum of pneumococcal disease ranges from local infections such as acute otitis media, acute rhinosinusitis and pneumonia, to invasive infections including meningitis and sepsis. In all these diseases, the pathogenic pneumococci initially disseminate from the nasopharynx, a single common site of colonization and carriage [3,4].

Disease, however, represents an evolutionary dead-end for the pneumococcus, since transmission to a new host occurs only via respiratory secretions from the reservoir of bacteria colonizing the nasopharynx [5,6]. Clinical studies and experimental colonization in humans have revealed that different pneumococcal serotypes can colonize repeatedly and concurrently. Each carriage event is maintained for weeks to months before being cleared [3,7,8].

Pneumococcal colonization is particularly common in young children, with a peak prevalence of 55 percent in children 3 years old, declining to 8 percent of 10 year olds and an even smaller proportion of adults [4,9]. Carriage is not only more frequent in children, but is also prolonged. Multiple studies across 3 continents demonstrate a consistent 2-fold increase in the duration of a given pneumococcal colonization event in children compared to adults [10–12]. The mechanism for delayed pneumococcal clearance by infants is not clear, however. One proposed explanation for more efficient clearance with increasing age is the development of antipneumococcal antibodies following clearance of pneumococcal carriage. These anticapsular antibodies cannot be the sole mediator of acquired protection against pneumococci, however, as pneumococcal disease decreases in childhood for all serotypes at a similar rate, a finding that would not be expected if each serotype would need to be carried to generate type-specific anticapsular antibodies [13]. This analysis implies that non-serotype specific mechanisms are responsible for the faster clearance of pneumococcal colonization that occurs with increasing age.

The molecular mechanisms underpinning pneumococcal clearance have been studied in an adult mouse model that faithfully recapitulates multiple aspects of human carriage, including the duration of carriage [14]. Clearance of colonization is independent of the acute inflammatory response and neutrophil influx into the nasopharynx, and furthermore does not require the development of anticapsular antibodies [15,16]. Rather, clearance depends on the recruitment of macrophages into the airway lumen, a process that requires Th17 cell immunity and the expression and sensing of the monocyte chemoattractant chemokine (C-C motif) ligand 2, or CCL2 (MCP-1) [17–19]. Chemokine production and macrophage recruitment occur in response to sensing by pattern recognition receptors TLR2 and Nod2, [18,19] as well as the macrophage scavenger receptor MARCO [20]. It is unclear how these pathways that normally lead to clearance of colonization from an adult host are absent or altered in infant mice.

Here, we show that infant mice are delayed in clearing pneumococcal colonization, and that this prolonged carriage is accompanied by slower macrophage recruitment. We demonstrate that increased macrophage chemoattractant expression due to acquisition of the infant microbiota prevents the formation of a chemokine gradient, and that this lack of chemokine gradient delays macrophage recruitment and pneumococcal clearance.

## Results

### Pneumococcal carriage is prolonged in infant mice

**Fig 1:**
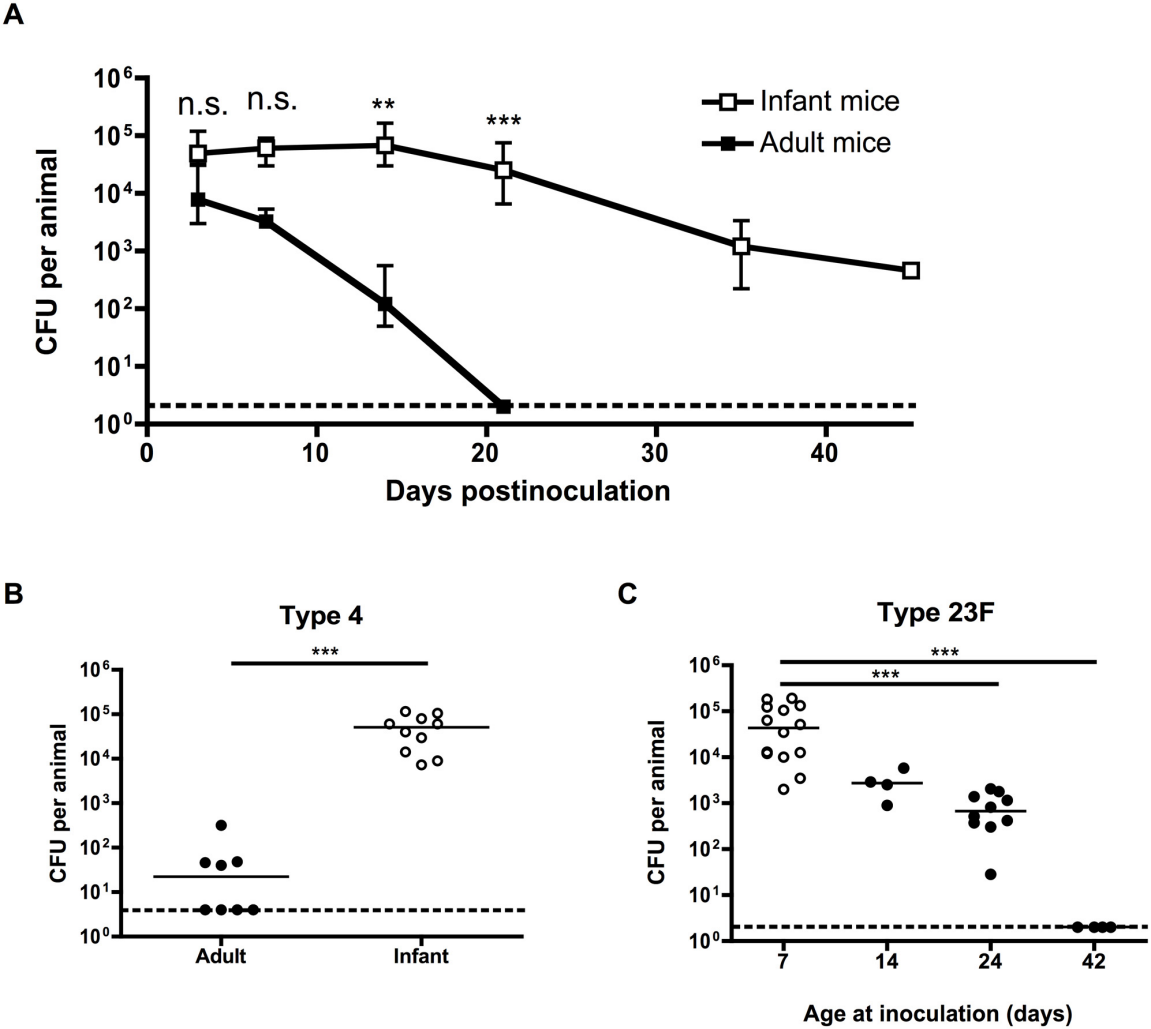
Pneumococcal carriage is prolonged in infant mice. (A) Adult (6 weeks old) and infant (7d old) mice were inoculated with pneumococcal strain P1121, and at the indicated number of days postinoculation (dpi), mice were sacrificed and nasal lavages obtained and plated to determine the load of colonizing pneumococci. (B) Adult and infant mice were inoculated with pneumococcal strain TIGR4 (type 4). Mice were sacrificed at 21 dpi and nasal lavages obtained and plated to determine the load of colonizing pneumococci. (C) Mice were inoculated with strain P1121 (type 23F) at different ages, ranging from 7 to 42 days. At 21 dpi, mice were sacrificed and nasal lavages obtained and plated to measure bacterial density. Points in (A) represent mean +/- SEM, with 5–18 mice per group. Horizontal lines indicate median values. Dotted lines indicate limit of detection. n.s. = not significant, ** = p < 0.01, *** = p < 0.001.

To determine whether pneumococcal carriage is prolonged in infant mice, adult (6 week old) and infant (7 day old) mice were intranasally inoculated with a pneumococcal isolate that does not cause systemic infection in mice (S1 Table) using a small volume and without anesthesia to prevent aspiration into the lower respiratory tract. At different timepoints, bacterial density was measured by plating nasal lavages. Adult mice started to clear colonization within a week after bacterial challenge, and had fully cleared pneumococci from the nasopharynx by 21 days postinoculation. By contrast, mice inoculated as infants maintained pneumococcal carriage for >6 weeks, and only started to clear colonization at 21 days postinoculation (Fig 1A). Delayed clearance in infant mice was not a strain-specific effect, as it was also seen with a clinical isolate of a different pneumococcal serotype (Fig 1B). The effect of age at inoculation waned over time, as seen by the gradual increase in clearance by 21 days postinoculation in mice inoculated at 7, 14, 24 or 42 days old (Fig 1C). Half of all mice inoculated as infants had cleared colonization by 45 days postinoculation, while half of all mice inoculated as adults cleared colonization completely at approximately 18 days postinoculation.

### Infant mice are impaired in macrophage recruitment during colonization

**Fig 2:**
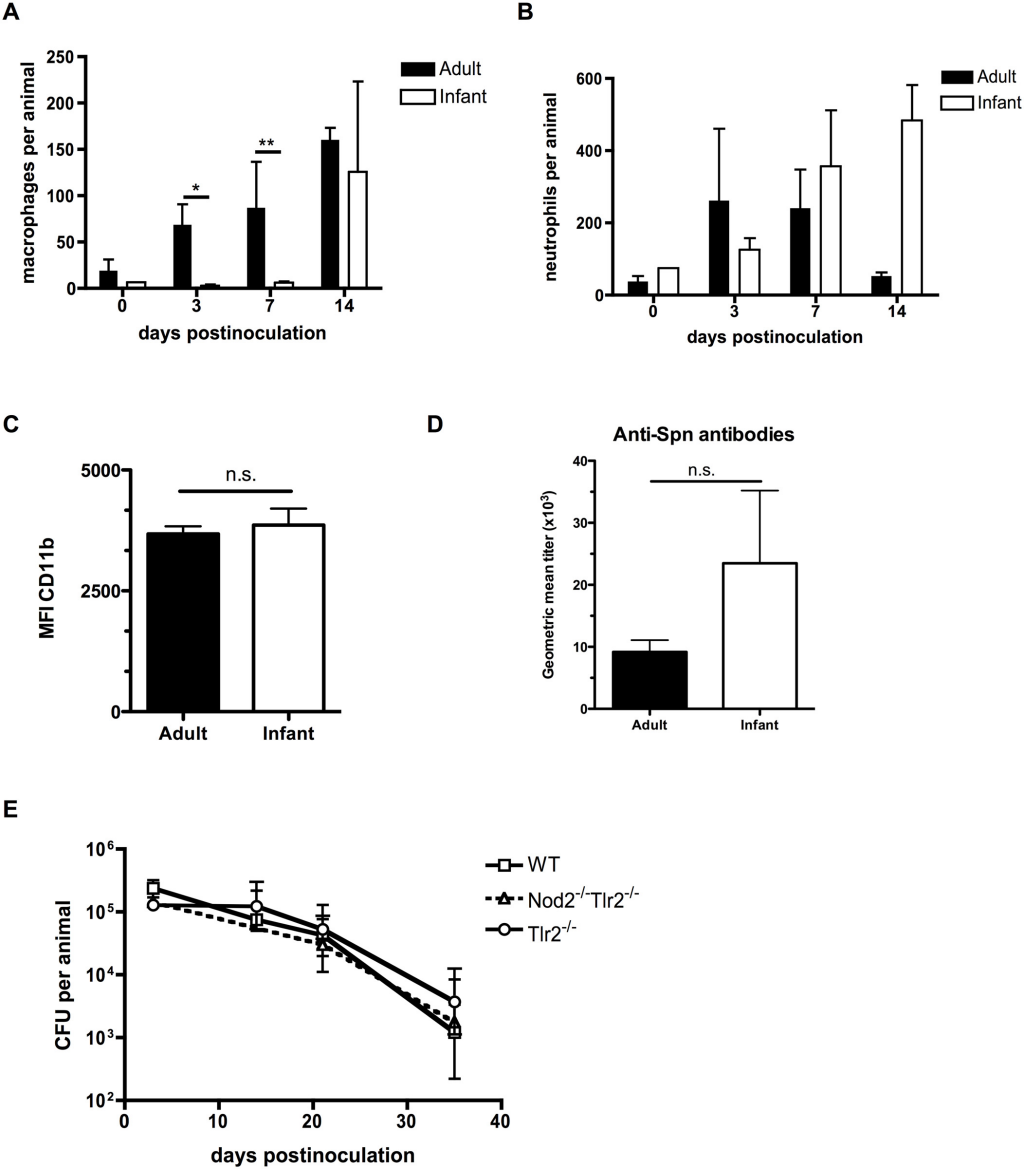
Infant mice are impaired in macrophage recruitment during colonization. (A-C) Adult (6 week old) and infant (7d old) mice were inoculated with strain P1121 for the indicated number of days. Nasal lavages were obtained and fixed and stained for flow cytometry to identify macrophages (F4/80+, CD11b-) and neutrophils (CD11b+, Ly6G+). CD11b surface expression was measured on myeloid cells and displayed as median fluorescence intensity. Samples represent at least 10 mice per timepoint. (D) Serum was obtained from adult and infant mice colonized with pneumococci for 21 days, or mock-colonized. Samples were analyzed by ELISA for the presence of antibodies specific to strain P1121. (E) Infant mice of the indicated genotype were colonized at 7d of age. Mice were sacrificed at the indicated timepoints and nasal lavages obtained and plated to measure bacterial load. Data are represented as mean +/- SEM. n.s. = not significant, * = p < 0.05, ** = p < 0.01.

Since pneumococcal clearance in adults is dependent on the sustained presence of macrophages into the nasopharynx, [18] we examined whether macrophages infiltrated the airway lumen of infant mice by using flow cytometry to quantify different cell populations in nasal lavages. Macrophage influx into the nasopharynx was delayed in infant mice compared to adults (Fig 2A). Inflammatory responses were not completely absent in infants, however, as the neutrophil influx into the nasopharynx in the first week post-inoculation was equivalent between adult and infant mice (Fig 2B). Myeloid cell maturation was not impaired in infant mice, as myeloid cells present in the nasal lavages from adult and infant mice had the same level of CD11b surface expression. (Fig 2C). Further evidence for a lack of age-related difference in macrophage maturation came from analysis of macrophages isolated from the peritoneal cavity of adult and infant mice. These had equal expression of surface receptors MHC class II, CD36 and MARCO, as well as the alternatively-activated macrophage polarization transcript *Rtnla* (S1 Fig). Furthermore, infant and adult mice were equally capable of mounting a humoral immune response to colonization, as measured by serum titers of antibodies specific to the colonizing strain of pneumococci (Fig 2D). In adult mice, the pattern recognition receptors Nod2 and TLR2 play redundant roles in macrophage recruitment and eventual clearance following pneumococcal colonization [19]. The infant clearance defect was epistatic with these pathways, as there was no additional delay in clearance of colonization in infant mice deficient in these pattern recognition receptors, implying that the infant clearance defect was redundant with these pathways (Fig 2E).

### Infant mice do not form a gradient of CCL2 expression during colonization

**Fig 3:**
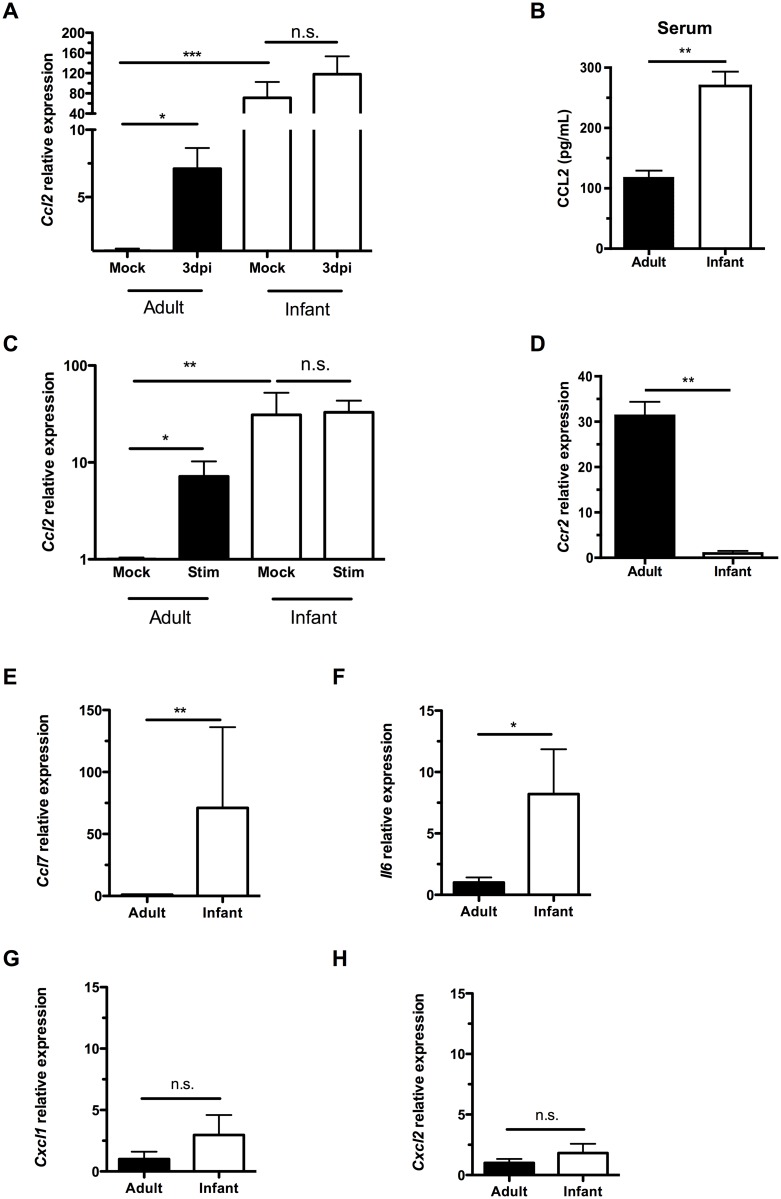
Infant mice do not form a gradient of CCL2 expression during colonization. (A) Adult (42d old) and infant (7d old) mice were inoculated with PBS (mock) or pneumococci. At 3d post-inoculation, nasal lavages were obtained with RLT lysis buffer, and RNA was isolated and reverse transcribed to cDNA. qRT-PCR was performed to measure relative expression of *Ccl2*. (B) Serum was obtained from adult and infant mice, and ELISA used to measure CCL2 levels. (C-D) Peritoneal macrophages from adult and infant mice were lysed with RLT buffer and RNA isolated. qRT-PCR was used to measure relative expression of *Ccl2* (C) and *Ccr2* (D). In (C), cultured macrophages were incubated overnight with PBS (Mock) or heat-killed bacterial lysates (Stim) prior to lysis. (E-H) Uncolonized adult and infant mice were sacrificed, and nasal lavages obtained with RLT lysis buffer. RNA was isolated and reverse-transcribed into cDNA. qRT-PCR was used to measure relative expression of *Ccl7*, (E) *Il6*, (F) *Cxcl1*, (G) and *Cxcl2* (H). Data are represented as mean +/- SEM. n.s., not significant. * = p < 0.05, ** = p < 0.01, *** = p < 0.001.

Previous work in adult mice demonstrated that induction of the monocyte/macrophage chemoattractant protein CCL2 (MCP-1) during colonization temporally correlated with clearance and occurs in macrophages in culture following exposure to pneumococci [19]. Colonized infants, however, did not upregulate *Ccl2* expression in the upper respiratory tract relative to expression in mock infants, as measured by qRT-PCR on RNA isolated from nasal lavages with RLT lysis buffer (Fig 3A). Due to the small volumes and dilution, it was not possible to reliably measure chemokine concentrations directly in lavage fluid. When directly comparing *Ccl2* expression in mock-infected adult and infant mice, baseline levels were significantly higher in the infant URT than the adult (Fig 3A). As with other chemokines, a concentration gradient of CCL2 is required to attract macrophages [21]. The lack of induction of *Ccl2* expression in infant mice during colonization suggested the concentration gradient of this macrophage-attracting chemokine was insufficient. Serum CCL2 levels were also elevated in infant mice compared to adults, potentially contributing to the failure to induce a concentration gradient from low CCL2 levels systemically to high levels at the site of colonization (Fig 3B).

We next wanted to identify the source of increased baseline CCL2 in infant mice. Since macrophages were not abundant in the nasal cavity, we turned to a distal site, the peritoneal cavity. We examined macrophage-intrinsic CCL2 signaling by eliciting macrophages from the peritoneal cavity with thioglycollate injection followed by peritoneal lavage 3 days later. Macrophages were purified by adherence, RNA was harvested from cells, and qRT-PCR performed. *Ccl2* expression was higher in infant macrophages than adults at baseline (Fig 3C). In the adult nasopharynx, stimulation by pneumococcal colonization led to increased *Ccl2* expression, while *Ccl2* expression in the infant nasopharynx did not increase above an already elevated baseline (Fig 3A). Macrophage-intrinsic *Ccl2* expression followed the same pattern. When stimulated with bacterial lysates, adult peritoneal macrophages increased *Ccl2* production, while infant peritoneal macrophages maintained the same elevated level of *Ccl2*, without further upregulation (Fig 3C). Therefore, cultured macrophages in isolation were sufficient to recapitulate the same pattern of CCL2 expression and upregulation as found in the nasopharynx. This tonic increase in *Ccl2* production by infant systemic macrophages was accompanied by a decrease in infant *Ccr2* production (Fig 3D).

Baseline expression of the related macrophage chemoattractant *Ccl7* (Fig 3E) and proinflammatory cytokine *Il6* (Fig 3F) were also elevated in infants compared to adults. In contrast, expression of the neutrophil chemoattractants *Cxcl1* (Kc) and *Cxcl2* (MIP2) was not elevated in the infant nasopharynx (Fig 3G and 3H). Together these findings suggested an inflamed state in the infant mucosa and that elevated serum and mucosal levels of macrophage chemoattractants in infants compromise the generation of a concentration gradient leading to the nasopharynx.

### CCL2 overexpression increases macrophage recruitment and pneumococcal clearance

**Fig 4:**
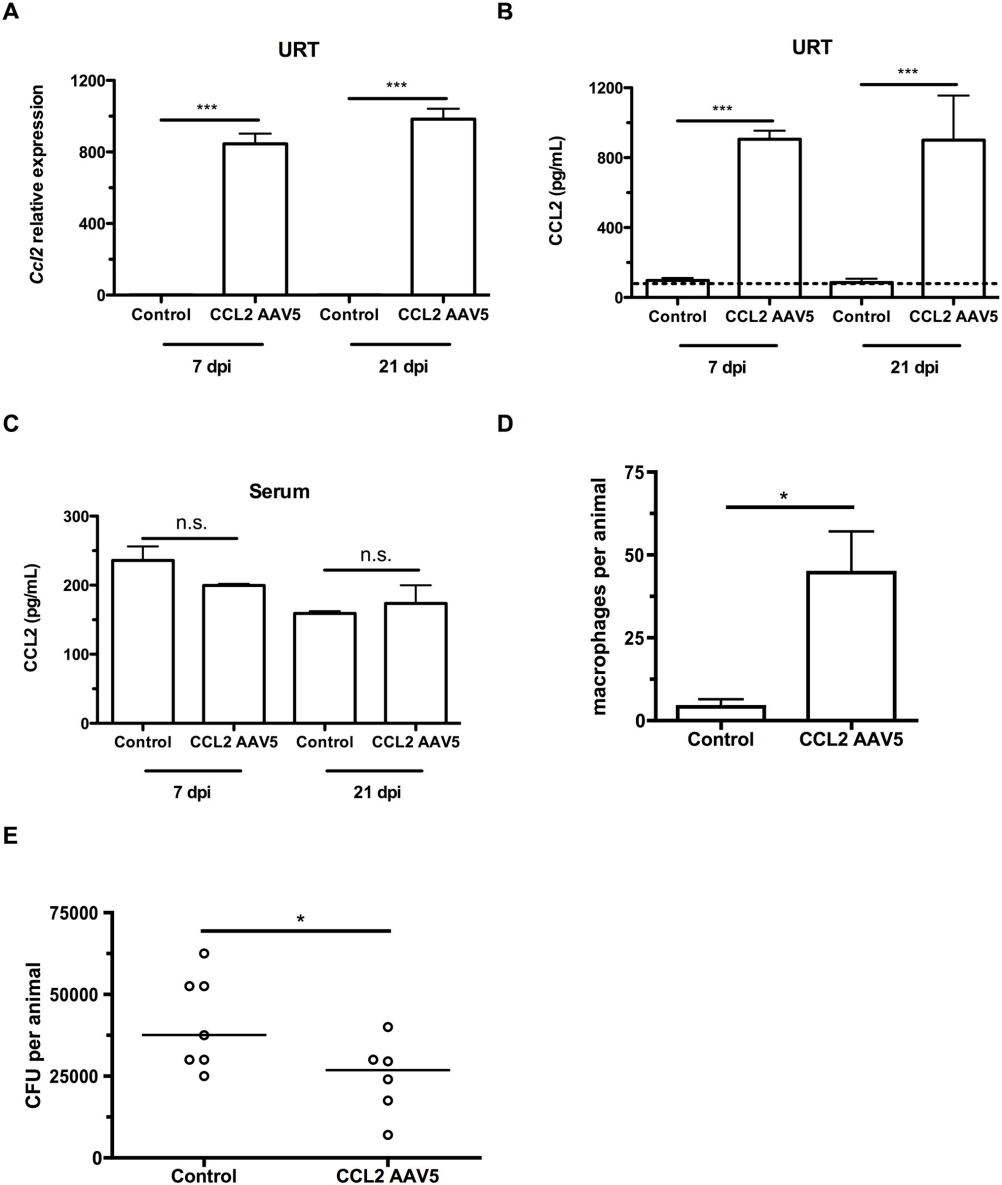
CCL2 overexpression increases macrophage recruitment and pneumococcal clearance. (A-C) Mice were inoculated with a control (GFP-expressing) or a CCL2-expressing AAV5 vector at 4d of age, followed by pneumococcal colonization at 7d old. Seven and 21 days later, mice were sacrificed and nasal lavages were obtained with RLT lysis buffer. (A) RNA was isolated and cDNA reverse-transcribed, followed by qRT-PCR to measure the relative expression of *Ccl2* in the upper respiratory tract, using primers *Ccl2*ORF-F and *Ccl2*ORF-R. ELISA was used to measure CCL2 protein levels in nasal lavage (B) and serum (C) at 7 and 21 dpi. (D) Mice were inoculated with a control (GFP-expressing) or a CCL2-expressing AAV5 vector at 4d of age, followed by pneumococcal colonization at 7d of age. Seven days later, mice were sacrificed, nasal lavages obtained and flow cytometry used to measure macrophage recruitment. (E) At 21d post-inoculation, nasal lavages were obtained and plated to measure the pneumococcal load in the nasopharynx. Data are represented as mean +/- SEM. n.s., not significant. * = p < 0.05, *** = p < 0.001. Dotted line, limit of detection.

To induce a concentration gradient of CCL2 in infant mice, we infected 4 day-old infant mice with adeno-associated viral (AAV) vectors expressing GFP (mock/vector control) or murine CCL2. Three days later, mice were colonized with pneumococci. At 7 and 21 days postinoculation, mice were sacrificed, nasal lavages obtained and flow cytometry performed to assess the macrophage influx into the nasopharyngeal lumen. CCL2 overexpression in the URT increased the local gradient in CCL2 concentration, as mice infected with the CCL2-expressing vector exhibited increased *Ccl2* transcription (Fig 4A) and CCL2 levels (Fig 4B), while CCL2 concentration in serum was unchanged (Fig 4C). CCL2 protein measurements in nasal lavage fluid are an underestimate, since lavage fluid is at least a 67-fold dilution of the fluid lining the nasal airway surface [22]. The impairment in macrophage recruitment in infant mice was partially recovered by ectopic CCL2 overexpression (Fig 4D). We assessed whether partial macrophage recruitment was sufficient to accelerate pneumococcal clearance by measuring colonization density at 21 days postinoculation, and found a small but significant recovery of the infant defect in clearance (Fig 4E).

### Depleting the microbiota limits infant *Ccl2* expression and accelerates pneumococcal clearance

**Fig 5:**
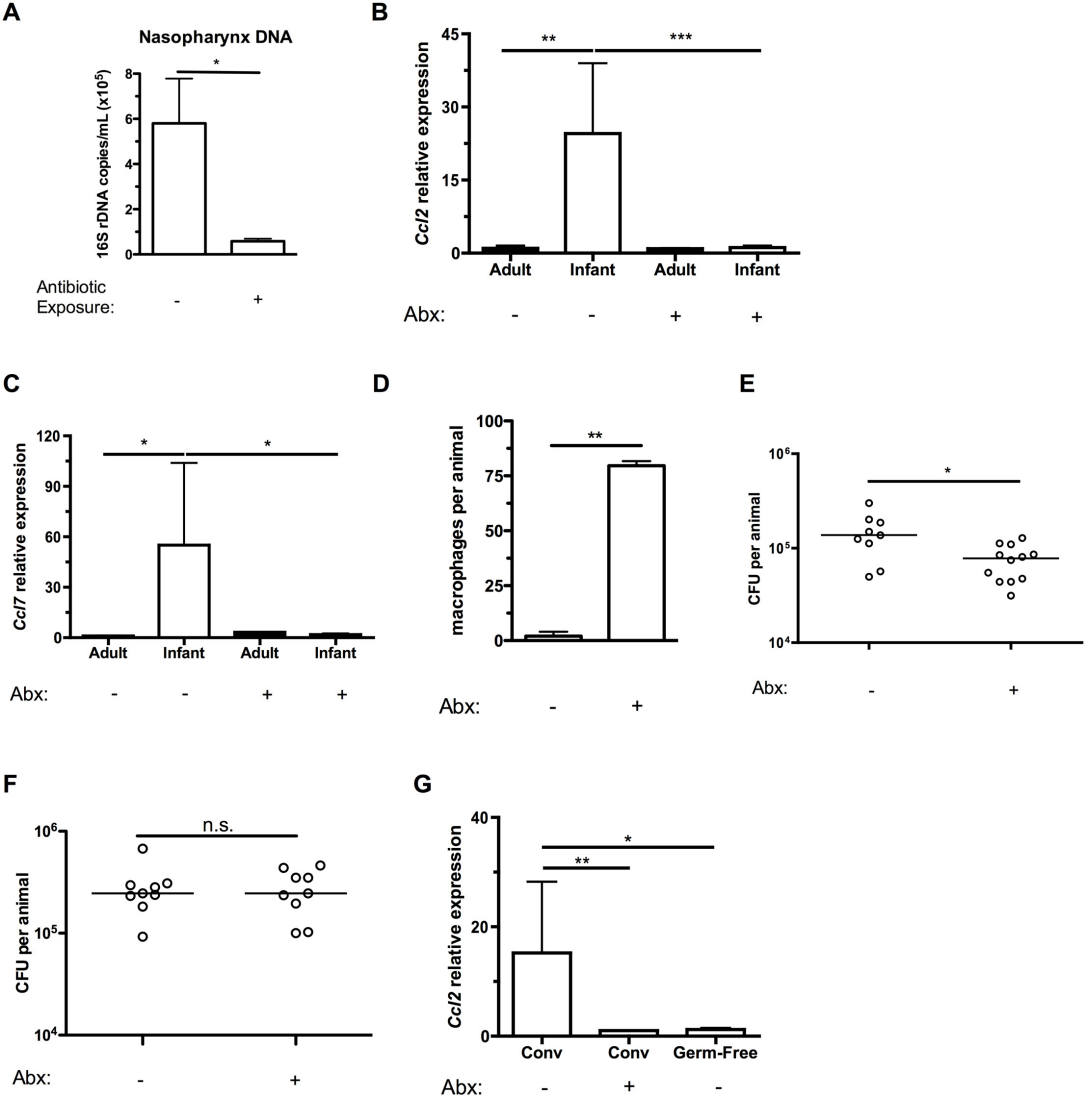
Depleting the microbiota limits infant *Ccl2* expression and accelerates pneumococcal clearance. (A) Breeding pairs were placed on sterile filtered tap water or water containing 5 antibiotics (Abx) for at least 5 days prior to giving birth, and continued until the infant mice were 10d old. Mice were sacrificed, and nasal lavages obtained with PBS. DNA was isolated from lavage fluid, and qPCR used to measure the number of copies of 16S bacterial ribosomal DNA. (B-C) Adult mice were maintained on sterile filtered tap water or water containing 5 antibiotics for at least 2 weeks. Breeding pairs were provided water as above. Mice were sacrificed, and nasal lavages obtained with RLT lysis buffer. RNA was isolated and reverse-transcribed into cDNA, and qRT-PCR used to measure relative expression of *Ccl2* (B) and *Ccl7* (C) in the nasopharynx. (D) Infant mice were exposed to tap water or antibiotics as in the previously described protocol. Treatments were continued until infant mice were 6d old, then all mice were given tap water to drink. 24 hrs later, mice were inoculated with pneumococci. At 7 dpi, mice were sacrificed and nasal lavages fixed and stained for flow cytometry to measure the number of macrophages present in the airway lumen. (E-F) Infant mice were treated as in (D). At 14d (E) and 7d (F) post-inoculation, mice were sacrificed. Nasal lavages were obtained and plated on media containing neomycin and catalase to measure pneumococcal density in the URT. (G) *Ccl2* expression was measured in the nasopharynx of tap- or antibiotic-water exposed conventionally-reared (Conv), or germ-free infant mice. Data are represented as mean +/- SEM. Horizontal lines indicate median values. n.s., not significant. * = p < 0.05, ** = p < 0.01, *** = p < 0.001.

We next sought to explain the elevated CCL2 expression in infants that was associated with higher baseline *Ccl2* expression in the URT, delayed macrophage recruitment and persistent pneumococcal colonization. Among the changes infants experience during normal development is the acquisition of a stable microbiota [23]. We examined whether the microbiota contributed to C-C motif chemokine expression by treating the drinking water of mice with antibiotics to deplete the flora. Adult mice were directly exposed to antibiotic-treated drinking water, while infant mice were exposed indirectly by treating the water of the dams from which the infants nursed. This indirect exposure was sufficient to decrease the commensal flora of the infant upper respiratory tract (Fig 5A). The magnitude of the depletion of the URT flora was consistent with the decrease in gut microbiota previously found in indirectly exposed infants [24]. Antibiotic treatment had no effect on URT expression of *Ccl2* in adults, but decreased baseline infant *Ccl2* expression to adult levels (Fig 5B). The microbiota was also responsible for the elevated infant baseline levels of *Ccl7* (Fig 5C). Limiting baseline C-C motif chemokine expression in the URT of infants allowed for normal responses to pneumococcal colonization, as macrophages were recruited into the nasopharynx of antibiotic-treated infant mice following 7 days of pneumococcal colonization, unlike tap-water treated mice (Fig 5D). Prior antibiotic treatment accelerated pneumococcal clearance, even 15 days after antibiotics were discontinued (Fig 5E). Even though antibiotics were removed from the drinking water starting 24 hrs before pneumococcal challenge in these experiments, it was still possible that any residual antibiotics could have direct effects on pneumococcal density in the nasopharynx. To exclude this possibility, we measured bacterial load at 7 days postinoculation, before the onset of clearance, and found no effect of antibiotics (Fig 5F). Nasopharyngeal expression of *Ccl2* was suppressed in germ-free infants compared to tap-water treated infants, confirming the microbiota was responsible for tonically elevated *Ccl2* expression in infants (Fig 5G).

## Discussion

Pneumococcal colonization and disease are more common in children than adults, but the mechanism underlying this predisposition has not been clear. Here, we demonstrated that an infant mouse model of carriage recapitulates the human delay in pneumococcal clearance. Using this model, we found that infant mice have delayed macrophage responses during colonization, which correlated with a failure to upregulate CCL2 signaling. Infant mice had tonic CCL2 production in the URT, indicating a compromised chemokine gradient. Reestablishing a gradient by ectopic overexpression of CCL2 partially restored macrophage recruitment and contributed to pneumococcal clearance. We found that the microbiota contributed to tonic macrophage chemoattractant expression, as depleting the commensal flora lowered expression of CCL2 and CCL7, restored normal macrophage responses and accelerated clearance of pneumococcal colonization. This effect was apparent even 14 days after stopping antibiotic treatment.

Higher pneumococcal loads in the infant nasopharynx have been previously reported, [25] but prior work did not examine innate immune responses in vivo that could explain delayed bacterial clearance, such as macrophage recruitment or expression of a CCL2 concentration gradient. Another study found delayed pneumococcal clearance in elderly mice, which correlated with decreased monocytic phagocyte recruitment and an increased inflammatory state at baseline in elderly mice [26]. This study did not find a role for elevated CCL2 expression in aged mice, however [26]. There is a growing understanding that overly exuberant inflammatory responses can be found both early and late in life, both in humans and mice [27]. Our observation of increased IL-6 expression in the infant URT is consistent with a more pro-inflammatory milieu early in life. It would be important to determine whether alterations in macrophage chemoattractant signaling are a consequence of this more generalized inflammatory state.

We found tonically elevated CCL2 expression in the URT of infant mice, which was dependent on the presence of the microbiota in infant mice. It was not clear whether the effect was restricted to infants due to the recent acquisition, size or composition of the flora in infant mice, or a unique response to the flora of the infant host. The mechanism by which the infant URT responds to the presence or acquisition of the microbiota by increasing production of CCL2, CCL7 and other inflammatory mediators remains unknown. Signaling events in the URT could reflect sensing of the local airway flora, or of commensals at distal sites such as the gut. The infant gut in mice is porous until weaning, [28] which could promote leakage of microbial products outside the containment of the gut lumen into otherwise sterile sites. Constitutive intestinal epithelial NF-κB activity is present in infant mice, and may be associated with endotoxin tolerance [29]. The flora has been shown to systemically prime innate immune responses in both adults [30] and newborns [24]. Our finding that CCL2 levels were elevated in infant serum and in infant macrophages isolated from a sterile site without a local commensal flora of its own, the peritoneal cavity, suggested a systemic effect of the flora on the proinflammatory environment of infants. Alternatively or additionally, sensing of microbial products that stimulate inflammation in infants may occur locally at the site of commensal colonization.

We also found macrophage recruitment to the infant murine nasopharynx was delayed during pneumococcal colonization. There is precedent for this pattern in humans as well, as the number of macrophages recruited to the nasal lumen during URT infections increased with age [31]. This effect, moreover, was independent of the number of prior infections [31]. CCL2 signaling in the human infant airway has not been studied, but there is some evidence for altered CCL2 production. One study found that serum CCL2 levels in normal children were higher than those found in normal adults, [32] consistent with our findings of elevated serum CCL2 in infant mice.

Inducing a gradient of CCL2 by local overexpression in the infant URT partially rescued the defect in macrophage recruitment. This partial recovery was associated with an increase in clearance of pneumococcal colonization. The incomplete recovery may have been due to continued tonically elevated expression of a related macrophage chemoattractant, CCL7 (MCP-3). This chemokine can also bind CCR2, the receptor for CCL2, and both it and CCL2 have additive functions in monocyte homeostasis and recruitment during infection [33,34]. In our study, the microbiota stimulated tonically high expression of both CCL2 and CCL7 in the infant nasopharynx. Together, these data suggest that simultaneous overexpression of CCL7 in addition to CCL2 and possibly other signals could lead to adult-like levels of macrophage recruitment, potentially fully accelerating pneumococcal clearance. CCR2 expression was appropriately suppressed considering the elevated CCL2 levels in infant macrophages, which indicated that the infant defect in CCL2 signaling was not a failure to respond to the ligand.

Altered monocyte/macrophage trafficking and CCL2 signals could be particularly important in mediating infant susceptibility to other infections, such as those with *Listeria monocytogenes*, which require both CCL2 and recruited monocyte-derived cells for clearance [35]. Infections in infancy are commonly caused by encapsulated bacteria, including opportunistic pathogens that colonize the URT, like the pneumococcus [36]. The delayed clearance of colonization in infant mice resembles tolerance, the failure to respond to an antigen. Elevated inflammatory pathways that cannot be further upregulated could be a mechanism for such tolerance. As a result, the mechanisms described here may reflect a general defect in infant innate immune responses and extend beyond pneumococcal carriage to clearance of other mucosal agents.

## Materials and Methods

### Ethics statement

This study was conducted according to the guidelines outlined by the Public Health Service Policy on the Humane Care and Use of Laboratory Animals. The protocol was approved by the Institutional Animal Care and Use Committee, University of Pennsylvania Animal Welfare Assurance Number A3079-01, protocol number 803231.

### Mice

C57Bl/6 mice were obtained from Jackson Laboratory. Germ-free mice were bred and raised in the Penn Gnotobiotic Mouse Facility at the University of Pennsylvania. Procedures were carried out according to an animal protocol approved by the University of Pennsylvania IACUC. For antibiotic treatment, tap water was supplemented with 0.5 g/L ampicillin (Sigma), neomycin (Calbiochem), gentamicin (Invitrogen) and metronidazole (Sigma), as well as 0.25 g/L vancomycin (Santa Cruz Biotechnology), then sterile-filtered. Water was changed every 4–5 days. Mice were sacrificed by CO_2_ inhalation and cardiac puncture.

### Bacterial strains and colonization

Pneumococcal strains used were the clinical isolates TIGR4 (capsule type 4), [37] P1547 (capsule type 6A) and P1121 (capsule type 23F, which is avirulent when injected into the murine bloodstream) [7]. For mouse colonization, pneumococci were grown in tryptic soy broth at 37°C until mid-log phase, then resuspended in sterile PBS. Mice were colonized with doses shown to be sufficient to establish high density colonization, 2x10^3^ CFU for infants and 1x10^7^ CFU for adults [38]. Pilot experiments using the adult dose in both infants and adults showed similar effects on clearance, macrophage recruitment and *Ccl2* expression. Mice were sacrificed at indicated timepoints, and nasal lavages obtained with 200 μL sterile PBS, as previously described [19]. Lavages were diluted onto TS agar with catalase (5,000 U/plate) (Worthington Biochemical) and 5 μg/mL neomycin added for quantitative culture overnight at 5% CO_2_.

### Flow cytometry

Nasal lavages were fixed in 2% paraformaldehyde, and then stained with antibodies to identify macrophages and neutrophils: anti-Ly6G (clone 1A8), anti-CD11b and anti-F4/80. Samples were run on a FACS Calibur instrument (Becton Dickinson) and analysis performed using FlowJo software (Tree Star).

### ELISA

For measurements of anti-pneumococcal antibody titers, pneumococcal strain P1121 was grown and resuspended to an OD_620_ of 0.1 in coating buffer (0.015 M Na_2_CO_3_, 0.035 M NaHCO_3_), then plated onto Immulon 2HB 96-well plates (Thermo) at 4°C overnight. Plates were washed with 0.05% Brij in PBS, and blocked for 1 hr at 37°C in 1% BSA in PBS. After additional washes, serum samples were added in serial 2-fold dilutions (made in 1% BSA in PBS) and incubated overnight at 4°C. Anti-pneumococcal antibodies were detected by incubating for 1.5 hrs at room temperature with an alkaline phosphatase-conjugated goat anti-mouse IgG antibody, followed by developing for 1 hr at 37°C with *p*-nitrophenyl phosphatase. Absorbance was measured at 415 nm. The sample dilution at which the absorbance equaled 0.1 was used to calculate the geometric mean titer. For measurements of CCL2 protein levels in serum and nasal lavages, an ELISA kit was used according to the manufacturer’s protocol (eBioscience).

### qRT-PCR

RNA was obtained from URT epithelium by lavage with RLT buffer (Qiagen) with 1% β-mercaptoethanol, or from cultured peritoneal macrophages by lysing cells in RLT buffer with 1% β-mercaptoethanol and frozen at -80°C until used. An RNeasy kit (Qiagen) was used to isolate RNA, and cDNA reverse transcribed by the High-Capacity cDNA Reverse Transcription kit (Applied Biosystems). qRT-PCR reactions were performed with Sybr Green (Applied Biosystems) with 10 ng cDNA and 0.5 μM primers. The ΔΔC_T_ method was used to compare conditions. Primer sequences were as follows: *Gapdh*-F 5’-AGG-TCG-GTG-TGA-ACG-GAT-TTG-3’; *Gapdh*-R 5’-TGT-AGA-CCA-TGT-AGT-TGA-GGT-CA-3’; [39] *Ccl2*-F 5′-AGC-TCT-CTC-TTC-CTC-CAC-CAC-3′; *Ccl2*-R: 5′-CGT-TAA-CTG-CAT-CTG-GCT-GA-3′; [19] *Ccl7*-F 5’-GCT-GCT-TTC-AGC-ATC-CAA-GTG-3’; *Ccl7*-R 5’-CCA-GGG-ACA-CCG-ACT-ACT-G-3’; *Il6*-F 5’-AGT-TGC-CTT-CTT-GGG-ACT-GA-3’; *Il6*-R 5’-TCC-ACG-ATT-TCC-CAG-AGA-AC-3’; [40]; *Cxcl1*-F 5’-CTG-GGA-TTC-ACC-TCA-AGA-ACA-TC-3’; *Cxcl1*-R 5’-CAG-GGT-CAA-GGC-AAG-CCT-C-3’; [41] *Cxcl2*-F 5’-CCA-CCA-ACC-ACC-AGG-CTA-C-3’; *Cxcl2*-R 5’-GCT-TCA-GGG-TCA-AGG-GCA-AA-3’; *Ccl2*ORF-F 5’-TTA-AAA-ACC-TGG-ATC-GGA-ACC-AA-3’; *Ccl2*ORF-R 5’-GCA-TTA-GCT-TCA-GAT-TTA-CGG-GT-3’; *Ccr2*-F 5’-GGT-CAT-GAT-CCC-TAT-GTG-G-3’; *Ccr2*-R 5’-CTG-GGC-ACC-TGA-TTT-AAA-GG-3’ [42]

### Peritoneal macrophages

Macrophages were obtained by injecting adult and infant mice with thioglycollate, followed 3 days later by peritoneal lavage with cold sterile PBS. Cells were spun down and resuspended in DMEM + 10% FBS. Cells were counted and adjusted to equal concentrations, then plated on 24-well non-tissue culture treated plates. After 2 hrs to allow macrophages to adhere, wells were washed 3 times and then media added back. Cells were used for RNA isolation after an overnight incubation, with or without stimulation with heat-killed bacterial lysates (10^7^ CFU pneumococci in 100 microliters heated to 65°C for 30 min, with an aliquot plated to verify complete killing).

### AAV

For overexpression, an AAV vector with the capsid from serotype AAV5 was used that expressed the open reading frame of murine CCL2, or GFP for the vector control, under the control of the chicken-beta actin promoter (Vector BioLabs, catalog # AAV-254826 for CCL2, 7006 for GFP). Vectors were concentrated to ~10^13^ GC/mL, and each mouse was inoculated with 10^11^ GC of vector.

### 16S rDNA quantification

DNA was extracted from 100 μL nasal lavage samples using the ZR Soil Microbe DNA Miniprep kit according to manufacturer’s instructions (Zymo Research). 16S rDNA copy number was measured using qPCR with a standard curve with a Topo vector containing *Escherichia coli* 16S rDNA (courtesy of Dr. Frederic Bushman). Reactions were performed using primers, probe and conditions as previously described [43].

### Statistical analysis

Comparisons were made using Prism software (Graphpad). Comparisons between groups for colonization data were made by Mann-Whitney U-test or Kruskal-Wallis test with Dunn’s posttest for two and three or more groups, respectively. All other comparisons were made by unpaired t-test or 1-way ANOVA with Newman-Keuls posttest for two and three or more groups, respectively.

## Supporting Information

S1 Table Strain P1121 is avirulent in infant mice. Seven day old mice were colonized intranasally with 2x10^3^ CFU pneumococci. Strains used were Type 23F, the strain P1121 used in experiments in the main text and a capsule type that is avirulent in adult mice, and Type 6A, a strain known to cause invasive infection in adult mice. Seven days later, mice were sacrificed, and equivalent volumes of blood diluted onto TS agar with catalase and neomycin for quantitative culture overnight at 5% CO_2_ for identification of pneumococci. Mice with any pneumococcal colonies isolated from blood were considered bacteremic.
(PDF)

S1 Fig Phenotypic characterization of adult and infant macrophages. Peritoneal macrophages were obtained from adult and infant mice. (A) Peritoneal macrophages were stained with antibodies to identify and characterize live macrophages: anti-CD45, anti-CD11b, anti-F4/80 and anti-MHCII. Samples were run on a FACS Canto instrument (Becton Dickinson) and the median fluorescence intensity of MHCII in live macrophages graphed using FlowJo software. (B-D) Peritoneal macrophages from adult and infant mice were counted and adjusted to equal concentrations, then plated on 24-well non-tissue culture treated plates. After 2 hrs to allow macrophages to adhere, wells were washed 3 times and media added back. After an overnight incubation, cells were lysed in RLT lysis buffer, RNA obtained and qRT-PCR performed to measure relative expression of surface scavenger receptors *Cd36* (B) and *Marco* (C), as well as the alternatively-activated macrophage-associated gene *Retnla* (D). Primer sequences were as follows: *Cd36*-F 5’-GAG-CAA-CTG-GTG-GAT-GGT-TT-3’; *Cd36*-R 5’-GCA-GAA-TCA-AGG-GAG-AGC-AC-3’; *Marco*-F 5’-GGC-ACC-AAG-GGA-GAC-AAA-3’; *Marco*-R 5’-TCC-CTT-CAT-GCC-CAT-GTC-3’; *Retnla*-F 5’-CCA-ATC-CAG-CTA-ACT-ATC-CCT-CC-3’; *Retnla-*R 5’-ACC-CAG-TAG-CAG-TCA-TCC-CA-3’.
(PDF)
