# Supplementary material for: Clearance of Pneumococcal Colonization in Infants Is Delayed through Altered Macrophage Trafficking
Source: PLoS Pathog. 2015 Jun 24;11(6):e1005004. doi: 10.1371/journal.ppat.1005004 (PMC4479461; doi:10.1371/journal.ppat.1005004)
Supplement: S1 Table — Seven day old mice were colonized intranasally with 2x10^3^ CFU pneumococci. Strains used were Type 23F, the strain P1121 used in experiments in the main text and a capsule type that is avirulent in adult mice, and Type 6A, a strain known to cause invasive infection in adult mice. Seven days later, mice were sacrificed, and equivalent volumes of blood diluted onto TS agar with catalase and neomycin for quantitative culture overnight at 5% CO2 for identification of pneumococci. Mice with any pneumococcal colonies isolated from blood were considered bacteremic. (PDF) [file ppat.1005004.s001.pdf]

Table S1

Pneumococcal strain:	Number of bacteremic mice	% bacteremic mice
**Type 23F (avirulent)**	0/8	0%
**Type 6A (virulent)**	6/9	67%

Strain P1121 is avirulent in infant mice. Seven day old mice were colonized intranasally with $2x10^3$ CFU pneumococci. Strains used were Type 23F, the avirulent strain P1121 used in experiments in the main text, and Type 6A, a strain known to cause invasive infection in adult mice. Seven days later, mice were sacrificed, and equivalent volumes of blood diluted onto TS agar with catalase and neomycin for quantitative culture overnight at 5% CO_2 for identification of pneumococci. Mice with any pneumococcal colonies isolated from blood were considered bacteremic.
