# Supplementary material for: Clearance of Pneumococcal Colonization in Infants Is Delayed through Altered Macrophage Trafficking
Source: PLoS Pathog. 2015 Jun 24;11(6):e1005004. doi: 10.1371/journal.ppat.1005004 (PMC4479461; doi:10.1371/journal.ppat.1005004)
Supplement: S1 Fig — Peritoneal macrophages were obtained from adult and infant mice. (A) Peritoneal macrophages were stained with antibodies to identify and characterize live macrophages: anti-CD45, anti-CD11b, anti-F4/80 and anti-MHCII. Samples were run on a FACS Canto instrument (Becton Dickinson) and the median fluorescence intensity of MHCII in live macrophages graphed using FlowJo software. (B-D) Peritoneal macrophages from adult and infant mice were counted and adjusted to equal concentrations, then plated on 24-well non-tissue culture treated plates. After 2 hrs to allow macrophages to adhere, wells were washed 3 times and media added back. After an overnight incubation, cells were lysed in RLT lysis buffer, RNA obtained and qRT-PCR performed to measure relative expression of surface scavenger receptors Cd36 (B) and Marco (C), as well as the alternatively-activated macrophage-associated gene Retnla (D). Primer sequences were as follows: Cd36-F 5’-GAG-CAA-CTG-GTG-GAT-GGT-TT-3’; Cd36-R 5’-GCA-GAA-TCA-AGG-GAG-AGC-AC-3’; Marco-F 5’-GGC-ACC-AAG-GGA-GAC-AAA-3’; Marco-R 5’-TCC-CTT-CAT-GCC-CAT-GTC-3’; Retnla-F 5’-CCA-ATC-CAG-CTA-ACT-ATC-CCT-CC-3’; Retnla-R 5’-ACC-CAG-TAG-CAG-TCA-TCC-CA-3’. (PDF) [file ppat.1005004.s002.pdf]

Figure S1

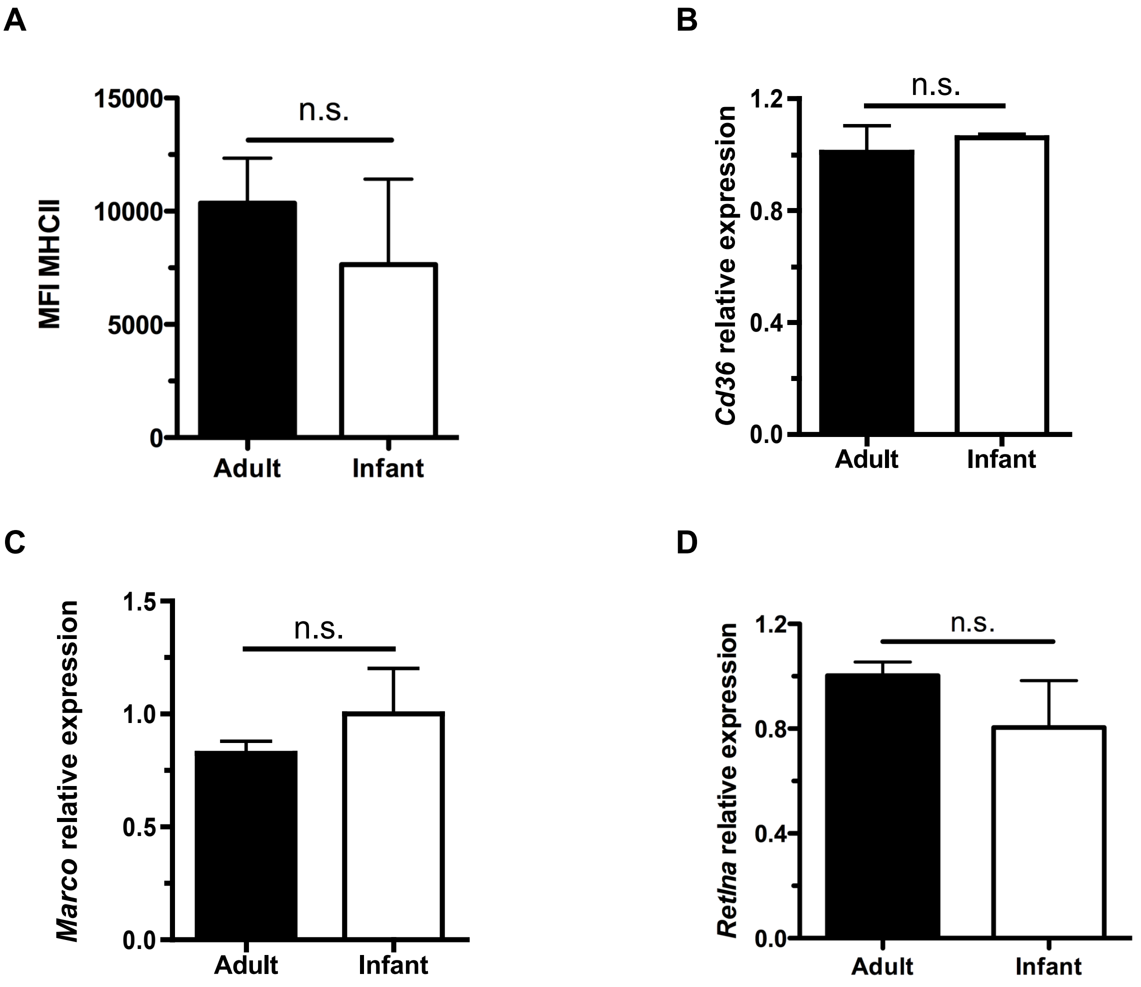

Phenotypic characterization of adult and infant macrophages. Peritoneal macrophages were obtained from adult and infant mice. (A) Peritoneal macrophages were stained with antibodies to identify and characterize live macrophages: anti-CD45, anti-CD11b, anti-F4/80 and anti-MHCII. Samples were run on a FACS Canto instrument (Becton Dickinson) and the median fluorescence intensity of MHCII in live macrophages graphed using FlowJo software. (B-D) Peritoneal macrophages from adult and infant mice were counted and adjusted to equal concentrations, then plated on 24-well non-tissue culture treated plates. After 2 hrs to allow macrophages to adhere, wells were washed 3 times and media added back. After an overnight incubation, cells were lysed in RLT lysis buffer, RNA obtained and qRT-PCR performed to measure relative expression of surface scavenger receptors *Cd36* (B) and *Marco* (C), as well as the alternatively-activated macrophage-associated gene *Retnla* (D). Primer sequences were as follows: *Cd36*-F 5'-GAG-CAA-CTG-GTG-GAT-GGT-TT-3'; *Cd36*-R 5'-GCA-GAA-TCA-AGG-GAG-AGC-AC-3'; *Marco*-F 5'-GGC-ACC-AAG-GGA-GAC-AAA-3'; *Marco*-R 5'-TCC-CTT-CAT-GCC-CAT-GTC-3'; *Retnla*-F 5'-CCA-ATC-CAG-CTA-ACT-ATC-CCT-CC-3'; *Retnla*-R 5'-ACC-CAG-TAG-CAG-TCA-TCC-CA-3'
